# A Novel Sprague-Dawley Rat Model Presents Improved NASH/NAFLD Symptoms with PEG Coated Vitexin Liposomes

**DOI:** 10.3390/ijms23063131

**Published:** 2022-03-15

**Authors:** Adil Farooq, Arfa Iqbal, Nosheen Fatima Rana, Misha Fatima, Tuba Maryam, Farhat Batool, Zahra Rehman, Farid Menaa, Shabia Azhar, Afrah Nawaz, Faheem Amin, Zuhair M. Mohammedsaleh, Salma Saleh Alrdahe

**Affiliations:** 1 Nanobiotechnology Lab, NUST Interdisciplinary Cluster for Higher Education (NICHE), School of Mechanical & Manufacturing Engineering (SMME), National University of Sciences and Technology (NUST), Islamabad 44000, Pakistan; adilfarooq.pg@smme.edu.pk (A.F.); aiqbal.bmes19smme@student.nust.edu.pk (A.I.); mfatima.bms20smme@student.nust.edu.pk (M.F.); tuba.bms20smme@student.nust.edu.pk (T.M.); farhat.bms20smme@student.nust.edu.pk (F.B.); zrehman.bms20smme@student.nust.edu.pk (Z.R.); sazhar.bmes19smme@student.nust.edu.pk (S.A.); anawaz.bmes18smme@student.nust.edu.pk (A.N.); 2 Department of Lnternal Medicine and Nanomedicine, California Lnnovation Corporation, San Diego, CA 92037, USA; menaateam@gmail.com; 3 School of Natural Sciences, National University of Sciences and Technology (NUST), Islamabad 44000, Pakistan; fahim.amin@sns.nust.edu.pk; 4 Department of Medical Laboratory Technology, Faculty of Applied Medical Sciences, University of Tabuk, Tabuk 71491, Saudi Arabia; zsaleh@ut.edu.sa; 5 Department of Biology, Faculty of Science, University of Tabuk, Tabuk 71491, Saudi Arabia; salrdahe@ut.edu.sa

**Keywords:** non-alcoholic fatty liver disease PEGylated vitexin loaded liposomal nanoparticles, lipogenesis inhibitor, vitexin, cirrhosis

## Abstract

Chronic liver disease (CLD) is a global threat to the human population, with manifestations resulting from alcohol-related liver disease (ALD) and non-alcohol fatty liver disease (NAFLD). NAFLD, if not treated, may progress to non-alcoholic steatohepatitis (NASH). Furthermore, inflammation leads to liver fibrosis, cirrhosis, and hepatocellular carcinoma. Vitexin, a natural flavonoid, has been recently reported for inhibiting NAFLD. It is a lipogenesis inhibitor and activates lipolysis and fatty acid oxidation. In addition, owing to its antioxidant properties, it appeared as a hepatoprotective candidate. However, it exhibits low bioavailability and low efficacy due to its hydrophobic nature. A novel rat model for liver cirrhosis was developed by CCL4/Urethane co-administration. Vitexin encapsulated liposomes were synthesized by the ‘thin-film hydration’ method. Polyethylene glycol (PEG) was coated on liposomes to enhance stability and stealth effect. The diseased rats were then treated with vitexin and PEGylated vitexin liposomes, administered intravenously and orally. Results ascertained the liposomal encapsulation of vitexin and subsequent PEG coating to be a substantial strategy for treating liver cirrhosis through oral drug delivery.

## 1. Introduction

Fibrosis delineates the substitution of damaged tissue by creating a collagenous scar [1]. It is followed by the continuance of healing responses that commence fibrogenesis and necroinflammation [1]. Liver cirrhosis is an advanced stage of fibrosis, characterized by histological growth of regenerative nodules surrounded by dense fibrotic septa, resulting in end-stage liver malady [1,2]. This fibrotic septum prevents regular oxygen supply and disturbs the blood exchange in the liver parenchyma. Such conditions consequently cause portal hypertension complemented with hepatocellular dysfunction [3]. Portal hypertension is characterized by certain clinical complications that correspond to variceal bleeding, peritonitis [4], ascites [5], and hepatic encephalopathy [6]. This advanced stage of fibrosis demonstrates anatomical changes like hepatocyte destruction, macro and microvascular transformation, nodule development, and formation of portosystemic shunts, etc. [3]. Cirrhosis is therefore termed as “advanced liver disease” or “chronic liver disease” (CLD) to accentuate the active progression and varying degree of disease prognosis from a histological perspective [2,6].

Liver cirrhosis is the 11th leading cause of death worldwide, accounting for 3.5% of all deaths [7,8]. It has an estimated steady upsurge of approximately 50 million incidences worldwide for the last two decades [3]. The non-alcoholic fatty liver disease (NAFLD), which is a manifestation of fat deposition on the liver, progresses to non-alcoholic steatohepatitis (NASH) [9]. It further advances to fibrosis (scarring) and ultimately to cirrhosis, leading to chronic disposition. The disease progresses due to some underlying mechanisms that do hepatocytes insults, such as viral hepatitis (B and C) [10], chemical injuries such as alcohol abuse [11], or a metabolic syndrome such as Wilson’s disease [12]. It is vital to identify the cirrhosis etiology to expect complications and layout of a treatment strategy. This can be done by histological and serological evaluations [2], termed as comprehensive metabolic panel (CMP) or a liver panel; Alkaline phosphatase (ALP), Alanine aminotransferase (ALT), Aspartate aminotransferase (AST), and Bilirubin to be more specific.

Clinically, cirrhosis is considered an end-stage disease that leads to death if liver transplantation is not performed [13]. Treatment options for cirrhosis include hepatocyte transplantation that ameliorates and reverses the liver physiology and advanced stage fibrosis [14,15]. In vitro expansion after isolating the progenitor or hepatocyte stem cells may have optimism in the transplantation paradigm [16,17]. Still, the proficiency of progenitor or stem cells is considerably low, and the essential manipulations for adequate engraftment in humans would sustain colossal risks of liver failure [18]. Similarly, genetic restoration by telomerase can boost hepatocyte regeneration [19], but increased telomerase activity may likely develop hepatocarcinogenesis [20].

One of the treatment options for cirrhosis reversal is the administration of antifibrotic agents. Research has been focused on the use of animal models to study the effects of antifibrotic agents on the reversal of cirrhosis [21]. The current murine models of cirrhosis include common bile duct ligation or hepatotoxin administration—alpha-naphthyl-isothiocyanate (ANIT), carbon tetrachloride (CCl4), allyl alcohol (AA), silica and 3,5-diethoxycarbonyl-1,4-dihydrocollidine (DDC). These models of cirrhosis do develop fibrosis and nodularity of the liver. Still, few models develop clinical complications that recapitulate the complete clinical spectrum of decompensated liver disease in human patients. These models showed the reversibility of putative antifibrotic agents or cessation of the agent responsible for liver damage [21]. Available antifibrotic therapies have focused on reducing hepatic inflammation rather than vanquishing fibrosis to stop or control the damage by keeping the underlying mechanism halted. Therefore, there is a need to develop an animal model that overcomes these limitations.

Vitexin, an 8-Dglucosyl-40, 5, 7-trihydroxy-flavone, has several pharmacological activities such as anticancer, antioxidant, anti-inflammatory, antihypertensive, antiviral, and antidepressant activity [22,23,24,25]. Recently, it has been reported to reduce liver inflammation in high-fat diet mice [26] and mice with acute ulcerative colitis [27]. Vitexin has also been reported to ameliorate abnormal lipid metabolism and reduce inflammatory cytokines in chronic stress NAFLD mice. Furthermore, it has been closely associated with suppressing lipid synthesis and TLR4/NF-κB signaling pathways to improve hepatic steatosis and inflammation [28]. Thus, vitexin can be a crucial antifibrotic candidate.

In this study, a novel model of liver cirrhosis is developed to observe the antifibrotic activity of vitexin in Sprague-Dawley Rats. Moreover, polyethylene glycol (PEG) coated vitexin liposomes (VLPs) were prepared, characterized, and realized for vitexin intravenous and oral delivery to treat cirrhosis in rats.

## 2. Materials and Methods

DPPC, cholesterol, commercially available vitexin, and PEG (Molecular weight 6000) were purchased from Sigma-Aldrich (MERK, Munich, Germany). Urethane and CCl4 were purchased from Strem Chemicals. Peanut oil (a delivery agent), ethanol (de-contaminant), and 10% neutral formaldehyde buffer (dissolved in PBS) were used. Sprague Dawley female rats were purchased from National Institute of Health (NIH), Islamabad.

### 2.1. Synthesis of VLPs

For the synthesis of liposomes, DPPC and cholesterol were used in 4:1 (percent molar ratio). At first, the lipid was weighed and dissolved in ethanol to form a 100 μM solution. 200 μM solution of vitexin drug was prepared in ethanol from which 500 μL drug solution was taken and mixed in lipid solution. The mixture was sonicated (at 80 MHz) for 40 min. Then 10 mL of water and lipid phase was allowed to be warmed in a water bath individually until the temperature reached 60 °C. The lipid phase was mixed with the water phase and this dispersion mixture was constantly mixed for ten minutes at 90 RPM. This new mixture was again sonicated (for 40 min at 50 MHz) and then allowed rotary evaporation (above the phase transition temperature i.e., 50 °C) to remove ethanol. Finally, the unentrapped drug was removed via mini column filtration (0.2 μm size) by centrifugation (at 4500 rpm) for 1 h [29].

### 2.2. Synthesis of PEG-VLPs

The same method that is used for the synthesis of VLPs was implicated for PEG-coated VLPs synthesis with few additional steps. Likewise, 100 μM solutions of lipid and 200 μM of vitexin drug were prepared in ethanol, from which 500 μL drug solution was taken and mixed in lipid solution. Following the sonication, the water phase was added to the lipid phase and a new solution was allowed to rotary evaporation. The next step was to cap the prepared VLPs, for which the mixture was diluted up to 50 mL; then 0.25% PEG was added dropwise, upon continuous stirring, and then allowed to rotary evaporation until 10 mL solution was left behind. The unentrapped drug was removed via mini column filtration by centrifugation (at 4500 rpm) for 1 h [30].

### 2.3. Physical Characterization

Characterization of VLPs and PEG-VLPs was done to evaluate and analyze their particle size, shape and surface charge, drug encapsulation, release efficiency, and dispersity Index.

#### 2.3.1. Spectroscopic Analysis and Fourier Transform Infrared Spectroscopy (FTIR)

UV-Vis spectra were measured by using Shimatzu UV-Vis 2800 BMS Scientific Technical Corporation (PVT) spectrophotometer (BMS Biotechnology Medical Services, Madrid, Spain), from 200–450 nm at a resolution of 1 nm. The de-ionized water was used as a reference for UV analysis. The UV spectra of Vitexin drug, blanks LPs, VLPs, and PEG-VLPs were recorded. For FTIR analysis, samples were allowed to air dry and then prepared using compressed KBr discs. FTIR spectra were recorded between 4000–350 cm^−1^ using Bruker FTIR Spectrophotometer ALPHA II (Westborough, MA, USA).

#### 2.3.2. Scanning Electron Microscopy

Both VLPs and PEG-VLPs were visualized by pouring a 200 μL sample on a coverslip using micropipette. The slide surface was then coated with gold in a sputter coater for 50 s at mA. Images were taken by using VEGA3 LMU Scanning Electron Microscope (Tescan, Czech Republic).

#### 2.3.3. Surface Charge, Zeta Potential, and Dispersity Index

Zeta potential (surface charge), size distribution, and dispersity of VLPs and PEG-VLPs were evaluated by Dynamic Light Scattering (DLS) using Malvern Zeta Sizer Ver. 7.12 (Malvern, UK).

### 2.4. Drug Encapsulation Efficiency

To find the congenial concentration of the vitexin for efficient encapsulation within liposomes, different dilutions of the vitexin were made and analyzed by UV spectrophotometer (200–450 nm) to obtain the possible linear standard curve. This standard curve value was further used in calculations to find out the unentrapped vitexin. Samples were centrifuged at 4500 rpm for 1 h, and supernatants were analyzed under UV-Vis. spectrometry to find unentrapped vitexin fractions [31]. After that, calculated values were used in the given formula.

Encapsulation Efficiency = (Total vitexin − Unentrapped vitexin)/Total vitexin × 100

### 2.5. Drug Release Efficiency

A total of 3 mL of VLPs and PEG-VLPs solutions were transferred separately into a 15 mL centrifuge tube and allowed for centrifugation for 10 min at 4500 rpm at 25 °C. On the other hand, 3 mL Phosphate buffer saline was added to VLPs and PEG-VLPs solutions. Following centrifugation, the supernatant was allowed for UV spectrophotometer analysis. Same procedure was followed after 1, 2, 4, 6, 12, 24, and 48 h. Analysis was carried out with an empty nanoparticle solution used as a control.

### 2.6. Development of Model

#### 2.6.1. Animals

60 Sprague-Dawley female rats weighing 85–105 g and ages of 4–6 weeks were used. The rats were kept under a 12-h light and dark cycle in separate cages with accessible water and food. The temperature was set around 27 °C with 60–70% humidity. Chloroform was used to anesthetize the rats to follow histological examination. Handling and caring of rats were centered upon the regulation of good laboratory practice issued by the US FDA (Food and Drug Administration) in 1978.

#### 2.6.2. Treatment Design

To evaluate the antifibrotic/anti-cirrhotic effects of VLPs and PEG-VLPs, diseased rats were categorized into different groups (Table S2). Body, liver weight and ascites were measured at the end of the experiment before dissection for histopathological and serological analysis.

#### 2.6.3. Liver Cirrhosis Induction

Initially, the rats were set free for one week. Normal rats were kept without exposing them to any adverse treatment in the entire procedure while diseased rats were exposed to detrimental chemicals via intraperitoneal injections. Additionally, 2.5% urethane was dissolved in Dimethyl sulfoxide (DMSO) and for the first two weeks, 1 mL/kg dose of urethane was injected intra-peritoneally twice a week. Then, CCl4 was mixed with pure peanut oil (50% *v*/*v*), and their intraperitoneal injections having the dose of 1 mL/kg were given twice a week for the rest of four weeks [32,33]. At least five rats were used per group.

### 2.7. Physical Parameters of Rats

Different conditions like food and water consumption, body weight, liver weight, and ascites were observed.

#### 2.7.1. Serological Indices

Blood was extracted from the heart for serological LFTs like AST, ALP, ALT, and T.B according to manufacturer’s guidelines.

#### 2.7.2. Histological Examination

After each week, one or two rats were sacrificed, and the liver, kidney, and spleen were harvested. The size, shape, texture, and color of diseased livers were obtained from liver tissue at the same time, fixed by 10% neutral-balanced formalin solution, 4 μm serial sections were obtained, followed by paraffin embedding. Subsequently, HE (Hematoxylin and Eosin) staining was carried out to observe the structural changes of hepatic tissue and hyperplasia of collagen fibers which can be observed in histopathological slides. The pathological grading was based on the criteria of Histological Grading and Staging for Fibrosis by NASH/NAFLD Clinical Research Network scoring system definition and scores [34], but some amendments were made according to variation in our histological observation (Table S2). Amendments were related to ‘Piecemeal Necrosis’ that include score = 1, 2, 3, 4 for Necro inflammation at Mild (few portal areas), Mild/moderate (most portal areas), Moderate (continuous around <50% of tracts or septa), Severe (continuous around >50% of tracts or septa) respectively [35]. Scoring was done by the METAVIR scoring system, which was developed in France in 1993, and has been adapted for the histological staging of liver disease in most etiologies of chronic liver disease [36,37].

## 3. Results

### 3.1. Physical Characterization of Vitexin Loaded—VLPs and PEGylated VLPs

**Figure 1:**
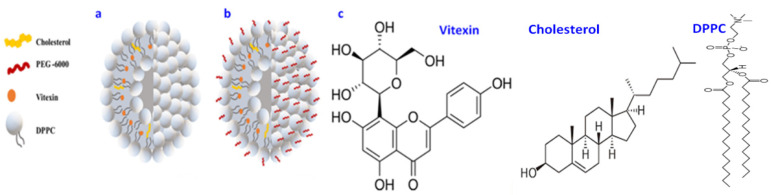
Pictorial depiction of (**a**) Vitexin liposomes (VLPs) (**b**) polyethylene glycol coated vitexin liposomes (PEG-VLPs) (**c**) Structure of Vitexin, Cholesterol, and Dipalmitoyl phosphatidylcholine (DPPC).

VLPs were prepared by the thin-film hydration method. For instance, Dipalmitoyl phosphatidylcholine (DPPC) and cholesterol were used to make a phospholipid bilayer vesicle. DPPC is a phospholipid (and lecithin). It consists of two C16 palmitic acid groups attached to a phosphatidylcholine head-group. The pictorial presentation of the liposomes and structure of vitexin is shown in Figure 1. After preparation, VLPs were coated by PEG, which led to broadened peaks.

#### 3.1.1. UV-VIS Absorption Spectroscopy

**Figure 2:**
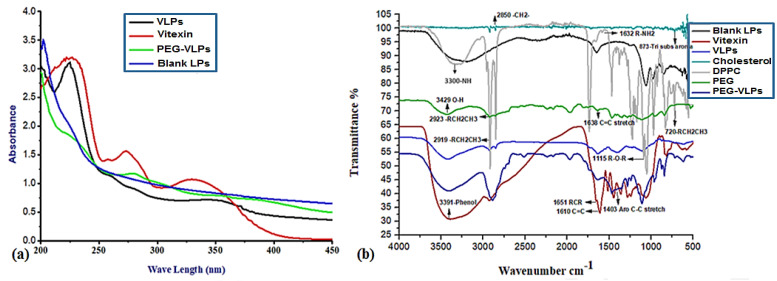
Comparative UV-Vis Spectra (**a**) and FTIR spectra (**b**) of Vitexin, Blank liposomes (LPs), Vitexin liposomes (VLPs), and polyethylene glycol coated vitexin liposomes (PEG-VLPs).

UV-VIS absorption spectroscopy of vitexin (Figure 2a) showed the absorbance peak at 330 nm. The blank liposomes (LPs) had peaks at 220 nm and 239 nm. VLPs showed high absorption at 225 nm and 340 nm, while PEG-VLPs showed absorption peaks at 270 nm. The shift in the peaks of VLPs to 270 nm and their further broadening were because of the PEG coating.

#### 3.1.2. Fourier Transform Infrared Spectroscopy (FTIR) Analysis

The FTIR spectrum (Figure 2b) of cholesterol indicated peaks or bands at 2850/cm (C.H. stretch, Alkanes), 873/cm (Tri-substituted Aromatics). DPPC spectrum showed peaks at 2919/cm (C.H. stretch, Alkanes), 1632/cm (R-NH2, Amines), 1115/cm (C-O stretch, Ether), 720/cm (RCH2CH3, Bending mode). PEG-6000 spectrum delineated peaks at 3429/cm (O-H stretch, Alcohol), 2923/cm (C.H. stretch, Alkanes), and 1638/cm (C=C stretch, Alkenes). Vitexin drug spectrum exhibited peaks at 3391/cm (Ar O-H bonded), 1651/cm (C=C stretch, ketone), 1610/cm (C=C stretch, Alkene), and 1403/cm (Ar C-C, Aromatics). In distinction, the spectrum of blank liposomes and PEG-liposomes depicted the disappearance of cholesterol’s 2850/cm (C.H. stretch, Alkanes) and 1632/cm (R-NH2, Amines) of DPPC. PEG-liposomes spectrum illustrated the disappearance of DPPC’s 1115/cm (C-O stretch, Ether). Vitexin’s 1651/cm (C=C stretch, Ketone) and 1403/cm (Ar C-C, Aromatics) has been disappeared in VLPs and PEG-VLPs spectra while DPPC’S 2919/cm (C.H. stretch, Alkanes) peak has been broadened. The observed changes in infrared bands proved the conformational arrangement in lipid biomolecules by incorporating with vitexin drug and PEG 6000.

#### 3.1.3. Particle Size and Area Distribution

**Figure 3:**
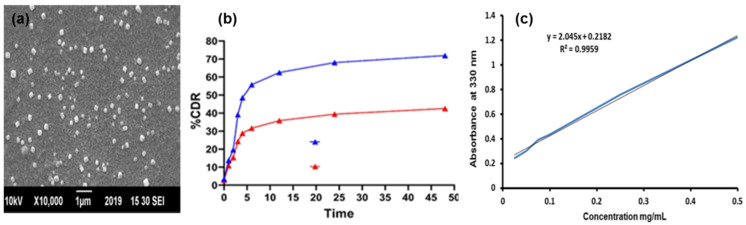
(**a**) SEM Image of vitexin liposomes (VLPs) and (**b**) Cumulative drug release from VLPs and polyethylene glycol coated vitexin liposomes (PEG-VLPs) (**c**) Standard curve.

The morphology and size of VLPs are visualized through Scanning Electron Microscopy (SEM) (Figure 3a). The image depicted the sphere-shaped nanoparticles with an average size of 155 nm.

The mean size of Blank LPs, VLPs, and PEG-VLPs s measured by Zeta sizer were 128 nm, 168 nm, and 458 nm, respectively. The average zeta potential of LPs, VLPs, and PEG-VLPs were −5.73 mV, −9.59 mV, and −0.3 mV, respectively, with a polydispersity index (PDI) of 0.488, 0.24, and 0.498, respectively. The use of PEG-6000 showed a notable increase in the zeta potential of VLPs, suggesting enhanced stability of VLPs.

#### 3.1.4. Drug Encapsulation Efficiency

The encapsulation efficiency was found to be 80%, delineating 80% entrapment of vitexin within liposomes.

#### 3.1.5. Drug Release Kinetics

The percentage cumulative drug release (CDR) was measured every two hours until 48 h. The release of vitexin from VLPs, and PEG-VLPs was 72% and 42%, respectively noted up to 48 h. This suggested the sustained release of drug with time from VLPs and PEG-VLPs (Figure 3b). Vitexin drug releases faster from VLPs than PEG-VLPs, hence improving the vitexin release profile.

### 3.2. Induction of Liver Cirrhosis in Sprague-Dawley Rats

The combination of CCL4_,_ urethane and peanut oil was used to induce cirrhosis. The histopathological, serological analysis, body and liver weight analysis, and ascites observations confirmed the successful induction of cirrhosis in rats.

#### 3.2.1. Histopathology of Liver, Kidney, and Spleen

**Figure 4:**
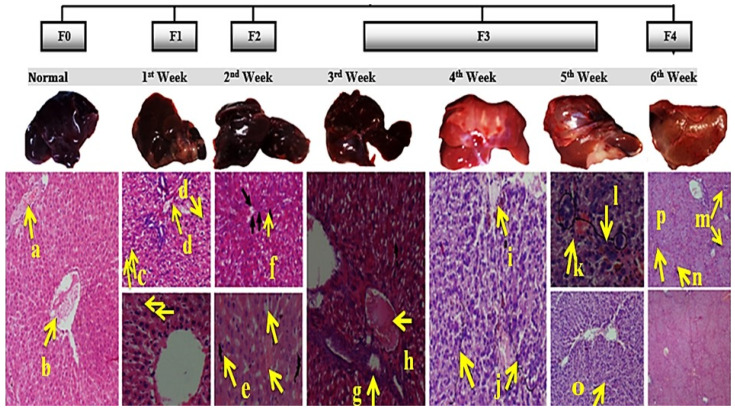
Meta-analysis of histological data in viral hepatitis (METAVIR) staging/classification for fibrosis and per week histopathology of the liver during cirrhosis induction. Normal (**a**,**b**), indications of mild fatty liver in first week (**c**,**d**), more fatty appearance in second week (**e**,**f**), severe fatty deposition in third week (**g**,**h**), Cholestasis in fourth week (**i**,**j**), and, complete cirrhosis achieved in fifth and sixth week (**k**,**l**).

The histopathology results confirmed the induction of cirrhosis after five weeks. The scoring was done to study the stages of liver cirrhosis (Table S1). In the first week (a, b in Figure 4) of cirrhosis induction, a 3/17 score was observed with mild fatty liver including mild portal fibrosis, balloon degeneration, and mild lobular inflammation (c, d in Figure 4). At week two, the score was further increased to 7/17 due to more fatty appearance fibrous portal expansion, congested sinusoids, and mild lobular inflammation (e, f in Figure 4). At week three, the score reached 8/17 with severe fatty deposition, congestion in sinusoids, and further portal expansion (g, h in Figure 4). At week four, cholestasis, liver parenchyma degeneration, and islands (i, j in Figure 4) were observed. At week five and six, complete cirrhosis was witnessed with the portal-to-portal bridging necrosis, balloon degeneration, and early to complete cirrhosis with scores 11–13/17 in different rats (k–p in Figure 4). These changes varied greatly from that of the normal liver histopathology results, thus validating the progression of fatty liver to cirrhosis in six weeks of induction.

The kidneys and spleen also presented the abnormalities associated with hepatotoxicity and cirrhosis (Supplementary material, Figure S1A,B).

#### 3.2.2. Serological Analysis

**Figure 5:**
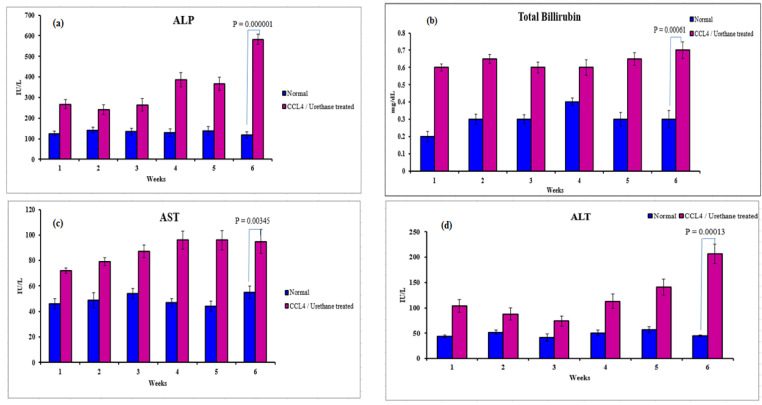
Serological indices of normal versus CCL4/urethane treated rats from week one to week six. Alkaline Phosphatase (ALP) (**a**), Total Bilirubin (TB) (**b**), Aspartate transaminase (AST) (**c**), Alanine transaminase (ALT) (**d**).

The serological analysis was conducted to check the functioning of the liver. It showed a significant difference in the liver functioning tests (LFTs) values obtained from the blood of the normal rats and the rats treated with CCl4 and urethane. This difference increased with the progression of weeks as noticed from Alkaline Phosphatase (ALP) (*p* = 0.00001) (Figure 5a), Total Bilirubin (TB) (*p* = 0.00061) (Figure 5b), Aspartate transaminase (AST) (*p* = 0.00345) (Figure 5c), and Alanine transaminase (ALT) (*p* = 0.00013) (Figure 5d), being most prominent in the sixth week indicating subsequent liver damage and cirrhosis development.

#### 3.2.3. Body, Liver Weight, and Ascites

**Figure 6:**
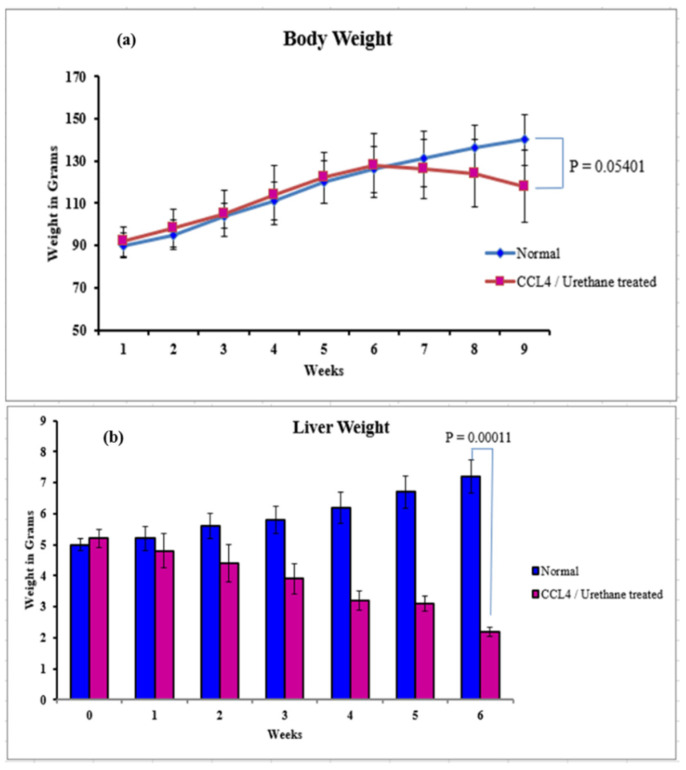
Weight analysis of normal versus CCL4/urethane treated rats from week one to week six. (**a**) Body (**b**) Liver. (Significance at *p* < 0.05).

The body and liver weight of rats treated with CCl4 and urethane gradually decreased with time compared to normal rats. Both body (*p* = 0.05401) and liver weight (*p* = 0.00011) significantly reduced in the sixth week of cirrhosis induction (Figure 6a,b). Weekly ascites level was checked according to scale organized for ascertaining the stage of liver cirrhosis (Table 1). No ascites were developed in the first two weeks after the start of induction. Mild ascites in the third week and moderate ascites were observed in the fourth and sixth week, whereas severe ascites were present in the fifth week.

### 3.3. Treatment of Cirrhosis Induced Sprague-Dawley Rats

The rats with successfully induced cirrhosis were subjected to treatment by vitexin (intravenous (IV) and oral gavage (OG)), VLPs (IV), and PEG-VLPs (OG)). The histopathological analysis, serological analysis, body and liver weight, and ascites observations showed the effectiveness of vitexin and PEG-VLPs in treating the cirrhosis-induced rat model by comparing these results with that of the normal rats.

#### 3.3.1. Histopathology Analysis of Liver, Spleen, and Kidney

**Figure 7:**
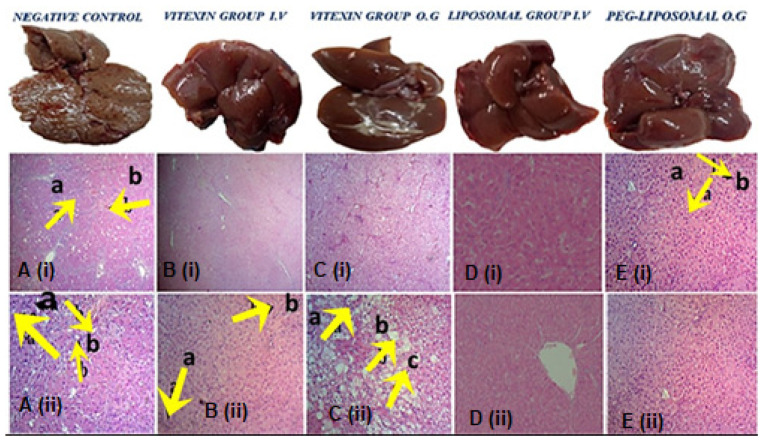
Liver histopathology of (**A**) Negative control, and after treatment with (**B**) Vitexin (intravenous (IV) treated group, (**C**) Vitexin Oral gavage (OG) treated group, (**D**) Vitexin liposomes (VLPs) IV treated group, (**E**) Polyethylene glycol coated vitexin liposomes (PEG-VLPs) OG treated group.

The liver, renal, and spleen histopathological analyses are provided in Figure 7, (Figures S2 and S3, Supplementary Material). In the liver tissue histology, the negative control group consisted of cirrhotic nodules and fibrous portal expansion ((i) in Figure 7A), liver cells dysplasia, and bile ductulus lined by atypical tall columnar cells ((ii) in Figure 7A). In the group treated with vitexin through IV, early cirrhosis stage ((i) in Figure 7B), liver cell dysplasia, and bile ductulus lined by atypical tall columnar cells ((ii) in Figure 7B) were observed. In the early cirrhotic stage, OG treated vitexin group showed piecemeal necrosis, marked fatty change, and feather degenerating hepatocyte ((i,ii) in Figure 7C). The VLPs IV treated group had histopathology within normal limits except dilated central vein ((i,ii) in Figure 7D). The PEG-VLPs OG treated group had prominent Kupffer cells, proliferating hepatocytes in and within normal limits ((i, ii) in Figure 7E).

In histopathology of renal tissues of vitexin IV treated group, kidneys’ glomeruli showed mesangial proliferation, 25–50% of cortical subcapsular tubules showed fatty change, necrosis, and vacuolar degeneration, 30% of medullary tubules also showed vacuolar degeneration, and blood vessels were congested. In the vitexin OG treated group, in kidney >20% of glomeruli were shrunken, blood vessels were congested, 20% degeneration in the medullary tubule, and 35% cortical damaged tubules (ATN) was observed. In VLPs IV treated group, renal histology showed the hypercellularity of glomeruli, atrophicity of glomeruli decreased (rare atrophic Glomeruli, i.e., <20%), <20% of tubules showed necrosis and degeneration, no cellular casts were observed, and regenerating tubules were seen. In the PEG-VLPs OG treated group, glomeruli had <10% trophicity, no cellular casts were seen, regenerating tubules were observed, and there was a lower extent of necrotic cells (Figure S2).

In spleen tissue histology of the vitexin, IV treated group, hyperplasia of white pulp, congestion, and hyperplasia of red pulp were observed. In the vitexin O.G treated group, germinal centers were prominent, and there was light fibrosis of red pulp but still decreased follicles and congestion of red pulp was seen. In VLPs IV treated group, follicles of variable sizes and increased in number with 50% of germinal centers were prominent. In PEG-VLPs treated group, there was no congestion and hyalinization, but still hyperplasia of red pulp was observed (Figure S3).

#### 3.3.2. Serological Analysis of Treated Mice

**Figure 8:**
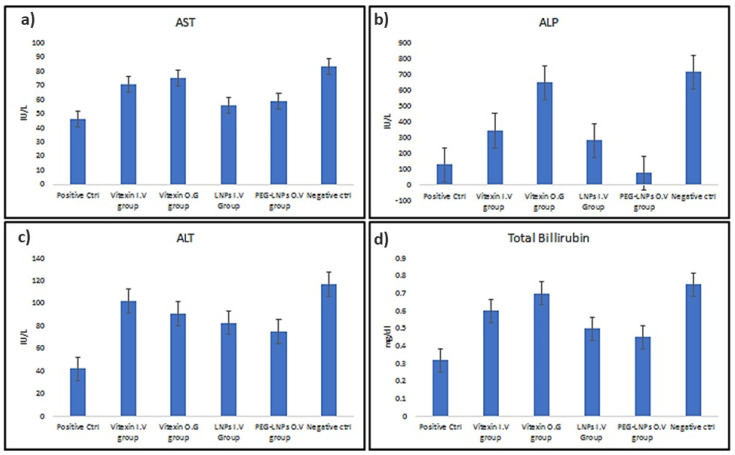
Serological indices of control versus diseased rats. Aspartate transaminase (AST) (**a**), Alkaline Phosphatase (ALP) (**b**), Alanine transaminase (ALT) (**c**). Total Bilirubin (TB) (**d**).

The serological indices noticed after performing LFTs showed a significant difference between normal/control and diseased rats (AST; *p* = 0.00116, T.B.; *p* = 0.00808, ALP; *p* = 0.000059, and ALT; *p* = 0.000399) (Figure 8). This indicated a prominent damage to the liver, causing cirrhosis. An almost similar trend with a comparatively lesser significant difference was noticed in the serological indices calculated from the blood of the rats administered vitexin through IV and OG (AST; *p* = 0.52259, T.B.; *p* = 0.03759, ALP; *p* = 0.00203, and ALT; *p* = 0.540678). Only the difference in the values of ALP showed that vitexin administered by OG proves to be more effective. In contrast, rest of the LFTs do not specify any considerable difference. Similarly, the difference in the values obtained of ALP (*p* = 0.000265) from the blood of rats administered intravenously with vitexin treated VLPs and orally with PEG-VLPs suggests that PEG-VLPs are the superior choice for the treatment of liver cirrhosis. Whereas AST (*p* = 0.32742), T.B. (*p* = 0.11078) and ALT (*p* = 0.540678) values did not alter much in the blood of rats administered intravenously with VLPs and orally with PEG-VLPs.

#### 3.3.3. Body Liver Weight and Ascites

**Figure 9:**
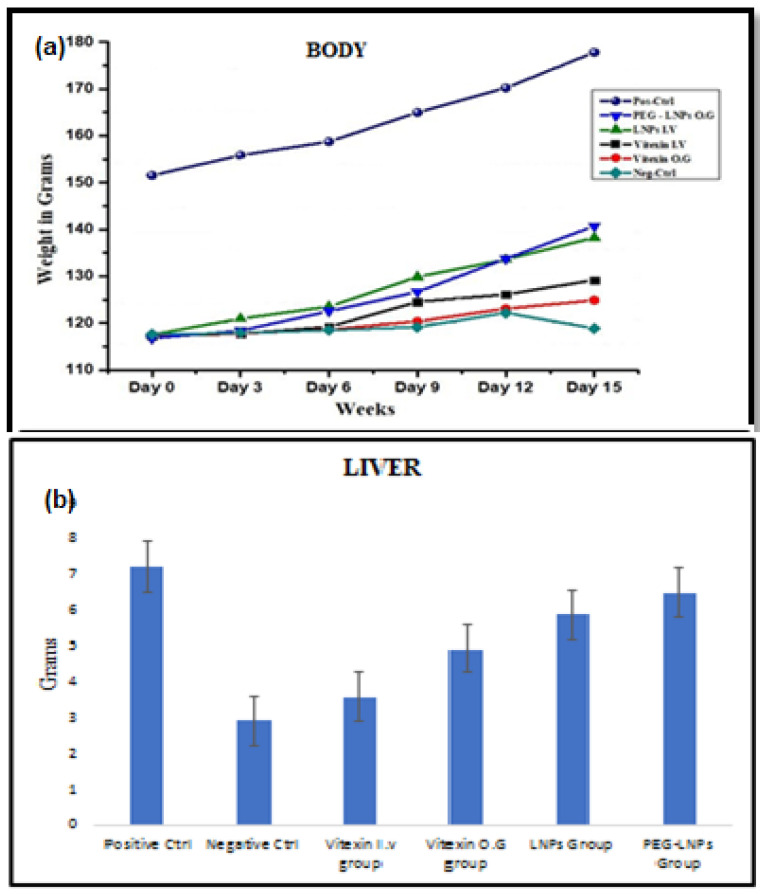
Weight Analysis of control and treatment groups (**a**) Body (**b**) Liver.

Body and liver weight analysis showed a significant decrease from that of the normal rats compared with the diseased rats (*p* = 0.00075 for body and *p* = 0.18252 for liver), indicating the successful development of liver cirrhosis (Figure 9a,b). The difference between the weight of rats treated intravenously and orally also varied considerably (*p* = 0.32007 for body and *p* = 0.00449 for liver), being of comparatively higher value in case of the oral administration, suggesting that vitexin administered by OG is more effective against liver cirrhosis. Weight reduction was seen the lowest in the group orally administered with PEG-VLPs and was also comparatively better than that of the rats intravenously administered with VLPs (*p* = 0.64807 for body weight and *p* = 0.02878 for liver weight).

Severe ascites were observed in the diseased rats of the negative control as compared to the normal rats. VLPs administered intravenously and orally showed moderate ascites, whereas mild ascites were seen in the case of VLPs. PEG-VLPs showed an absence of ascites (Table 2).

## 4. Discussion

Liver cirrhosis is generally considered a dynamic disorder bearing the advancing and regressive temperament. In the continuum of changes that characterize an advanced chronic liver disease, early diagnosis before decompensation is a significant step towards achieving mortality reduction. Various pharmacological and nonpharmacological methods are explored to prevent decompensation (an alarming move in this disease’s natural history) [3]. However, a potent antifibrotic drug is still awaited [38,39], and liver transplantation is the only curative alternative available.

Recently, vitexin was established to alleviate the non-alcoholic fatty liver disease via activation of AMP-activated protein kinase (AMPK) that resulted in lipolysis and inhibition of lipogenesis [40]. However, the low aqueous solubility and poor bioavailability of vitexin limits its therapeutic window [37,41,42]. With the advent of nanotechnology, various drugs associated with poor solubility and low bio-availabilities have been reformulated through carrier-driven entry mechanisms based on drug delivery systems [43]. In the present study, we successfully formulated the PEG-VLPs with the aim to treat liver cirrhosis. Vitexin, being hydrophobic, may encapsulate within the hydrophobic tails of the DPPC bilayer. To achieve optimized PEG-VLPs with desired features such as size, PDI, zeta potential, and encapsulation efficiency, different parameters like DPPC and Cholesterol ratio, drug to lipids ratio, sonication time and amplitude, and the temperature of water bath during rotary evaporation were monitored. The smaller size blank LPs were obtained with DPPC and cholesterol ratio of 4:1. The average size of blank LPs and VLPs were observed to be 128 nm and 190 nm, respectively. Drug release from VLPs was noted to be 72% after 48 h examination. Pegylation enhanced the steric repulsion and hence stabilized the formulation [44]. It was observed that 44% of the drug was released from PEG-VLPs, which delineated the sustained release as an erosion-controlled release mechanism [45]. In the present study, comparative FTIR of Vitexin, VLPs, and PEG-VLPs showed the involvement of different functional groups as a characteristic feature of various constituents involved in particle formation.

The preset research also reported a novel liver cirrhosis model by coadministration of CCL4/urethane. CCL4 is most commonly used hepatotoxin in cirrhosis induction in rodents. It mimics human chronic disease linked with toxic damage. The hepatic biotransformation of CCL4 produces trichloromethyl radical that induces several free radicals and lipid peroxidation reactions that contributes to an excruciating phase reaction characterized by the necrosis of centrilobular hepatocytes [46]. Furthermore, prolonged administration of urethane has also been reported to induce hepatic vein fibrosis [47]. The present study is one of its kind that reported coadministration of these chemicals and analyzed its effects on the liver, kidney, and spleen by histological examination along with liver function tests. After five weeks of urethane/CCL4 administration, the appearance of collagenous scars depicted successful induction of cirrhosis [46,47].

After successful induction of cirrhosis, the antifibrotic effects of vitexin, VLPs and PEG-VLPs were analyzed to validate the model. The histological, serological, body, and liver weight results exhibited the significant reversibility of collagenous scars to normal hepatic tissue paradigm by using the VLPs and PEG-VLPs contrary to the blank vitexin. Fatty changes in the kidney along with tubular necrosis were noticed that perpetuated from mild to severe over the period of five weeks. At the same time, lymphoid follicle formation, hyaline deposition, and subsequent hyperplasia of red pulp in spleen were indicators of successful cirrhosis model development by the end of sixth week.

Following treatment of the diseased rats, the histological, serological, body and liver weight results depicted significant improvement in group treated with PEG-VLPs. Apart from that, VLPs treated group also showed better results up to some extent. Thus, for the reversibility of collagenous scars to normal hepatic tissue, the use of the encapsulated vitexin drug is better choice, contrary to blank vitexin. Hence, liposomes encapsulation along with PEG coating is proved as the most effective stratagem in enhancing the bioavailability and pharmacokinetic behavior of vitexin drug. It has also proven the anti-fibrotic activity of considerable therapeutics against liver cirrhosis. In addition to the route of administration for blank drug, the intravenous route has showed better results, although further research is required to study the effects of oral administration. As such, VLP and PEG-VLPs are found to be effective in treatment of liver cirrhosis and are potential candidates for NAFLD/NASH disease management. Future studies are required to study gene/protein expression analysis of inflammatory biomarkers such as interleukin 6 (IL-6), tumor necrosis factor-alpha (TNF-α), transforming growth factor-beta (TGF-β), and monocyte chemoattractant protein 1 (MCP-1) to understand the mechanism of action of cirrhosis reversal by vitexin in detail.

## 5. Patents

“Vitexin Nano formulation for treatment of advance liver disease” is submitted to Intellectual Property Office of National University of Sciences and Technology Islamabad for National Patent.

## Figures and Tables

**Table 1:** Weekly comparison of Ascites of normal versus CCL4/urethane treated rats.

Weeks	0	1st	2nd	3rd	4th	5th	6th
**Ascites**	-	-	-	-	-	+	-
	-	-	-	-	+	+	+
	-	-	-	+	+	+	+
**No Ascites: - - -, Mild Ascites: - - +, Moderate Ascites: - + +, Severe Ascites: + + +**							

**Table 2:** Analysis of Ascites of normal versus treatment groups.

Normal	Negative Ctrl	Vitexin (I.V.)	Vitexin (O.G.)	LNPs	PEG-LNPs
**-**	**+ + +**	**+ +**	**+ +**	**+**	**-**
**No Ascites: - -, Mild Ascites: +, Moderate Ascites: + +,** **Severe Ascites: + + +**					

## Data Availability

Not applicable.

## Supplementary Materials

The following supporting information can be downloaded at: https://www.mdpi.com/article/10.3390/ijms23063131/s1.
