# Supplementary material for: A Novel Sprague-Dawley Rat Model Presents Improved NASH/NAFLD Symptoms with PEG Coated Vitexin Liposomes
Source: Int J Mol Sci. 2022 Mar 15;23(6):3131. doi: 10.3390/ijms23063131 (PMC8948922; doi:10.3390/ijms23063131)
Supplement: Supplementary file 1 [file ijms-23-03131-s001.zip › ijms-1434442-supplementary.pdf]

Supplementary Material

A novel Sprague-Dawley Rat model presents improved NASH/NAFLD symptoms with PEG coated vitexin liposomes

Adil Farooq[1], Arfa Iqbal[1], Nosheen Fatima Rana[1], Misha Fatima[1], Tuba Maryam[1], Farhat Batool[1], Zahra Rehman[1], Shabia Azhar[1], Afrah Nawaz[1], Fahim Amin[2], Zuhair M. Mohammed Saleh[3] and Salma Saleh Turki Alrdahe[3].

[1]Nanobiotechnology Lab NUST Interdisciplinary Cluster for Higher Education (NICHE), School of Mechan ical & Manufacturing Engineering (SMME), National University of Sciences and Technology (NUST), Islamabad 44000, Pakistan.mfatima.bms20smme@student.nust.edu.pk;nosheen.fatima@smme.nust.edu.pk;tuba.bms20smme@student.nust.edu.pk;farhat.bms20smme@student.nust.edu.pk;afarooq.bmes17smme@student.nust.edu.pk

[2]School of Natural Sciences, National University of Sciences and Technology (NUST), Islamabad 44000, Pakistan.

[3]Department of Medical Laboratory Technology, Faculty of Applied Medical Sciences, University of Tabuk, Tabuk 71491, Saudi Arabia. Zsaleh@ut.edu.sa

[4]Department of Biology, Faculty of Science, University of Tabuk, Tabuk 71491, Saudi Arabia. salrdahe@ut.edu.sa

*Correspondence: nosheen.fatima@smme.nust.edu.pk

Abstract: Chronic liver disease (CLD) is a global threat to the human population, with manifestations resulting from alcohol-related liver disease (ALD) and non-alcohol fatty liver disease (NAFLD). NAFLD, if not treated, may progress to non-alcoholic steatohepatitis (NASH). Furthermore, inflammation leads to liver fibrosis, cirrhosis, and hepatocellular carcinoma. Vitexin, a natural flavonoid, has been recently reported for inhibiting NAFLD. It is a lipogenesis inhibitor and activates lipolysis and fatty acid oxidation. In addition, owing to its antioxidant properties, it appeared as a hepatoprotective candidate. However, it exhibits low bioavailability and low efficacy due to its hydrophobic nature. A novel rat model for liver cirrhosis was developed by CCL4/Urethane co-administration. Vitexin encapsulated liposomes were synthesized by the 'thin-film hydration' method. Polyethylene glycol (PEG) was coated on liposomes to enhance stability and stealth effect. The diseased rats were then treated with vitexin and PEGylated vitexin liposomes, administered intravenously and orally. Results ascertained the liposomal encapsulation of vitexin and subsequent PEG coating to be a substantial strategy for treating liver cirrhosis through oral drug delivery.

Keywords: Non-alcoholic fatty liver disease PEGylated vitexin loaded liposomal nanoparticles; lipogenesis inhibitor; Vitexin; Cirrhosis

Steatosis		
Grade	Parenchymal involvement	Score
	<5%	0
	5-33%	1
	33-66%	2
	>66%	3
Inflammation		
Lobular inflammation	Assessment of all inflammatory foci	
	No foci	0
	<2 foci per X 200 field	1
	2-4 foci per X 200 field	2
	>4 foci per X 200 field	3
Portal inflammation	Assessed under low magnification	
	None to minimal	0
	Greater than minimal	1
Piecemeal Necrosis	Absent	0
	Mild (focal, few portal areas)	1
	Mild/moderate (focal, more portal areas)	2
	Moderate (continuous around <50% of tracts or septa)	3
	Severe (continuous around >50% of tracts or septa	4
Microgranulomas	Small aggregates of macrophages	
	Absent	0
	Present	1
Large lipogranulomas	In portal areas or adjacent to the central vein	
	Absent	0
	Present	1
Fibrosis Stage	Method of brunt	
	None	0
	Perivenular/perisinusoidal fibrosis	1
	Combined pericellular portal fibrosis	2
	Septal/bridging fibrosis	3
	Cirrhosis	4

Table S1. NASH/NAFLD Clinical Research Network Scoring system. (Definition and score).

Sr. No.	Group	Dosage	Duration
1.	Normal	---	6 weeks
2.	Negative Control Group/ diseased	---	6 weeks
3.	Vitexin treated intravenous (IV)	10mg/Kg	15 days
4.	Vitexin treated Oral Gavage (OG)	10mg/Kg	15 days
5.	Vitexin treated Liposomal nanoparticles (IV)	500µg/Kg	15 days
6.	PEGylated Vitexin treated Liposomal nanoparticles (OG)	500µg/Kg	15 days

Table S2. Treatment design-based animal groups.

1. Histopathology of spleen and kidney during 6 week period of cirhossis induction period

In the 1st week of induction, histological changes in the kidney showed tubular necrosis in 25% of tubules, glomeruli showed mild mesangial proliferation, unremarkable blood vessels, and <20% of atrophic glomeruli. Although, interstitium and medullary tubules appeared to be normal. In the spleen, hyperplasia of white pulp, germinal center increased in numbers, and mild red pulp hyperplasia was observed. In the 2nd week, architectural changes included tubular necrosis in 30% of tubules, 20% of glomeruli were atrophic, blood vessels were unremarkable, rare cellular casts and medullary interstitium showed congestion while germinal follicles were increased in numbers, the germinal center was prominent and red pulp showed congestion. In the 3rd week, kidney glomeruli showed hypercellularity, 40% of cortical tubules showed necrosis, the medullary tubule was within the normal limit, and blood vessels were congested. In the spleen, follicles were poorly demarcated, their center was not prominent, and red pulp

was unremarkable. In the 4th week, focal tubular necrosis, focal hyaline cast, few glomeruli showed necrosis (hypocellularity), and blood vessels were congested. In the spleen, follicles were normal in appearance but were ill-defined.

50% of germinal centers were not prominent. Blood showed sub-epithelial hyaline deposition and deposition of pinkish material in blood vessels. In the 5th week, 20% of glomeruli were shrunken, 20% tubule showed necrosis and hyaline casts, cortical cells showed degeneration, blood vessels were congested, and mild fatty change was observed in the kidney. While follicles were normal in number, the germinal centers were not prominent, and the red pulp showed shrinkage in the spleen. In the 6th week, glomeruli were atrophic with hypo-cellularity and hyper-cellularity. In some glomeruli, tubular necrosis with few casts, congested blood vessels, and moderate fatty changes were observed. While in the spleen, follicles were decreased in number, germinal centers were not prominent, and fibrosis of red pulp occurred.

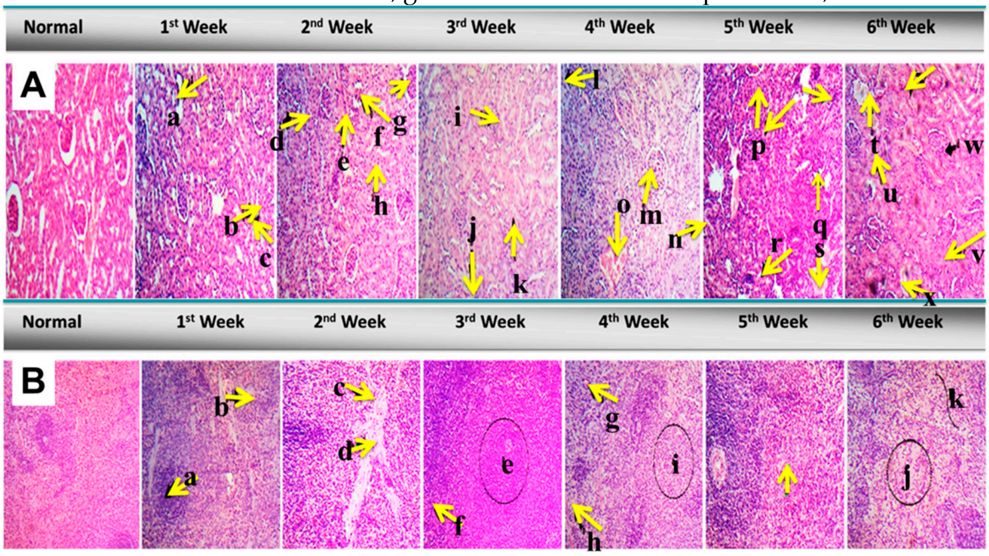

Figure S1. Histological changes in (A) kidneys and (B) spleen during cirrhosis induction. (A) The histopathology of kidney tissues in 1st week indicated atrophic glomerulus (a), tubular necrosis (b), dead tubular cells (c). In the 2nd week, tubular degeneration (d) and mild lobular inflammation (f) were observed. At the third week, interstitial inflammation (i), congested blood vessels (j), and tubular necrosis (k) were observed. At the 4th week, cast in tubules (l), hyper-cellularity in the glomerulus (m), tubular necrosis (n), and congested blood vessels were observed (o). In the 5th week, fatty change (p), atrophic glomerulus (q), acute tubular necrosis (ATN) (r), and congested blood vessels (s). In the 6th week, congested blood vessels (t), fatty change (u), ATN (v), hypercellularity (w), and cellular cast (x) were observed. (B) This histopathology of spleen tissues in 1st week indicated (a) Shrunken follicles and (b) Hyaline deposition. In the 2nd week, (c) Hyaline globule and (d) Hyperplastic thick trabecular were observed. The 3rd week was characterized by (e) poorly demarcated prominent lymphoid follicles and (f) unremarkable red pulp. At the 4th week, (g) red pulp hyperplasia containing red blood cells (RBCs) and (h) macrophages were observed, and (i) increased germinal follicles were also present. In the 5th week, hyperplasia in red pulp was observed, and at the 6th week (j), a hyalinization and fibrosis of red pulp and (k) increased hyperplasia of red pulp was observed.

2. Renal and spleen histology after treatment

2.1 Renal Histopathology

The renal histopathology (Figure 9) consisted of a) atrophic glomerulus, b) fatty change, c) vacuolar degeneration, and d) ATN (Acute tubular necrosis) in negative control. In intravenously treated vitexin group, a) atrophic glomerulus, b) hypercellularity, and c) regenerating tubule appeared. The orally treated vitexin group had a) lobulation of glomerulus b) atrio tubular necrosis (ATN). PEG-liposomal treated

group showed a) no cellular cast, b) regenerating tubules, and c) necrosis. In liposomal treated group the arrows show mild necrosis.

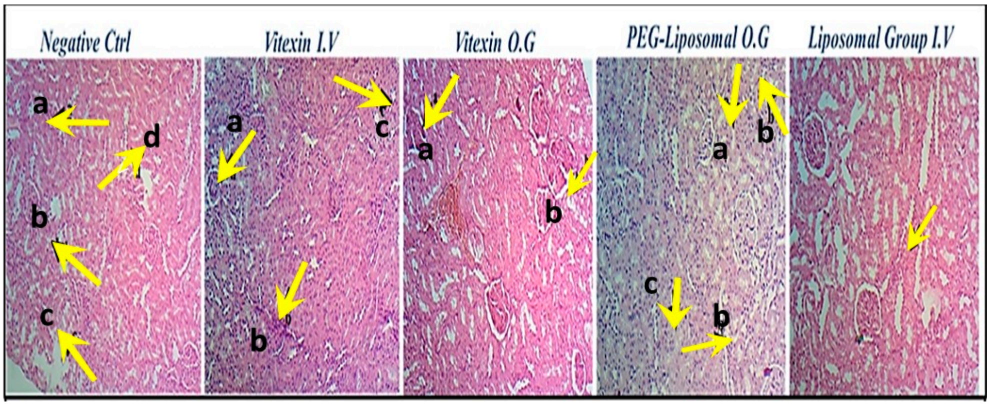

Figure S2. Kidney histopathology after treatment of (a) Negative ctrl, (b) Vitexin intravenous group, (c) Vitexin Oral Gavage group, (d) VLPs treated group, (e) PEG-VLPs treated group.

2.2. Spleen Histopathology

As shown in Figure 10., the negative control group of spleen histopathology showed a) red pulp with congested and active macrophages, b) hyperplastic thick trabeculae, c) giant cells, and d) fibrosis. The intravenously vitexin treated group showed a) fibrosis in red pulp and b) enlarged follicle. The orally vitexin treated group had a) hyperplasia of white pulp, b) congestion and hyperplasia of red pulp. Liposomal treated group show congestion with hyalinization of red pulp. PEG-liposomal treated group showed hyperplasia of red pulp and mild expansion in white pulp.

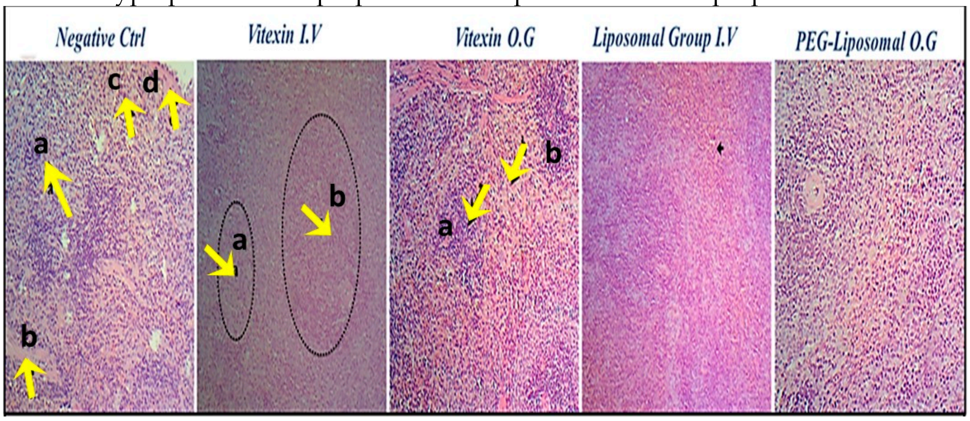

Figure S3. Spleen histopathology after treatment of (a) Negative ctrl, (b) Vitexin intravenous group, (c) Vitexin Oral Gavage group, (d) VLPs treated group, (e) PEG-VLPs treated group.
